# Supplementary material for: Single-exposure visual memory judgments are reflected in inferotemporal cortex
Source: eLife. 2018 Mar 8;7:e32259. doi: 10.7554/eLife.32259 (PMC5843407; doi:10.7554/eLife.32259)
Supplement: Source data 1. — Behavioral data include the forgetting function and reaction times, each as a function of n-back. Neural data include the spike count responses of each unit to the same images presented as novel and as familiar at a range of n-back (Source_data.zip). [file elife-32259-data1.zip › Source_data/Readme.rtf]

README for Source_data.zip
Meyer and Rust (2018) eLife.

Data.mat is a MATLAB file containing data collected and analyzed using the procedures described in Results and Methods. These data correspond to the pooled data across the two animals involved in the study.

FFmn: a 1 x 8 vector containing the average proportion chose familiar for each of the 7 n-back (1, 2, 4, 8, 16, 32, 64) and for novel images (Fig 6c, green).

RTmn_corr: a 1 x 8 vector containing mean reaction times on correct trials for each of the 7 n-back (1, 2, 4, 8, 16, 32, 64) and for novel images (Fig 6f, red).

RTmn_err: a 1 x 8 vector containing mean reaction times on error trials for each of the 7 n-back (1, 2, 4, 8, 16, 32, 64) and for novel images (Fig 6f, cyan).

NOVmat: a 799 unit x 107 array containing the spike count responses of each of 799 units to 107 novel images.

FAMmat: a 799 unit x 107 array containing the spike count responses of each of 799 units to the same 107 images contained in NOVmat but when the images were presented as familiar.

NBACK: a 1 x 10^7^ vector indicating the n-back separation between the novel and familiar presentations of each image in NOVmat and FAMmat.
