# Supplementary material for: Single-exposure visual memory judgments are reflected in inferotemporal cortex
Source: eLife. 2018 Mar 8;7:e32259. doi: 10.7554/eLife.32259 (PMC5843407; doi:10.7554/eLife.32259)
Supplement: Transparent reporting form [file elife-32259-transrepform.docx]

***eLife’s* transparent reporting form**

We encourage authors to provide detailed information *within their submission* to facilitate the interpretation and replication of experiments. Authors can upload supporting documentation to indicate the use of appropriate reporting guidelines for health-related research (see [EQUATOR Network](http://www.equator-network.org/%20)), life science research (see the [BioSharing Information Resource](https://biosharing.org/)), or the [ARRIVE guidelines](http://www.plosbiology.org/article/info:doi/10.1371/journal.pbio.1000412) for reporting work involving animal research. Where applicable, authors should refer to any relevant reporting standards documents in this form.

If you have any questions, please consult our Journal Policies and/or contact us: [editorial@elifesciences.org](mailto:editorial@elifesciences.org).

**Sample-size estimation**

- You should state whether an appropriate sample size was computed when the study was being designed
- You should state the statistical method of sample size computation and any required assumptions
- If no explicit power analysis was used, you should describe how you decided what sample (replicate) size (number) to use

Please outline where this information can be found within the submission (e.g., sections or figure legends), or explain why this information doesn’t apply to your submission:

No explicit power analysis was used. A description of sample size is included, “The sample size (number of successful sessions recorded) was chosen to approximately match our previous work (Pagan et al., 2013).”

**Replicates**

- You should report how often each experiment was performed
- You should include a definition of biological versus technical replication
- The data obtained should be provided and sufficient information should be provided to indicate the number of independent biological and/or technical replicates
- If you encountered any outliers, you should describe how these were handled
- Criteria for exclusion/inclusion of data should be clearly stated
- High-throughput sequence data should be uploaded before submission, with a private link for reviewers provided (these are available from both GEO and ArrayExpress)

Please outline where this information can be found within the submission (e.g., sections or figure legends), or explain why this information doesn’t apply to your submission:

Information about how often each experiment was performed is included in the Methods: ”Recording sessions began after the monkeys were fully trained on the task and after the depth and extent of IT was mapped within the recording chamber. Combined recording and behavioral training sessions happened 4-5 times per week across a span of 5 weeks (monkey 1) and 4 weeks (monkey 2).”

Replication: Our results highlight qualitative replication across different animals (Fig 3; Fig 7; Fig 8a-b).

We would not consider any of our data an outlier.

Criteria for inclusion / exclusion: Criteria for inclusion of individual recording sessions is described in the Methods, and the numbers of excluded session based on each criterion are indicated, “A multi-channel recording session was included in the analysis if: (1) the recording session was stable, quantified as the grand mean firing rate across channels changing less than 2-fold across the session; (2) over 50% of neurons were visually responsive (a loose criterion based on our previous experience in IT), assessed by a visual inspection of rasters; and (3) the number of successfully completed novel/familiar pairs of trials exceeded 100. In monkey 1, 21 sessions were recorded and 6 were removed (2 from each of the 3 criterion). In monkey 2, 16 sessions were recorded and 4 were removed (1, 2 and 1 due to criterion 1, 2 and 3, respectively).”

**Statistical reporting**

- Statistical analysis methods should be described and justified
- Raw data should be presented in figures whenever informative to do so (typically when N per group is less than 10)
- For each experiment, you should identify the statistical tests used, exact values of N, definitions of center, methods of multiple test correction, and dispersion and precision measures (e.g., mean, median, SD, SEM, confidence intervals; and, for the major substantive results, a measure of effect size (e.g., Pearson's r, Cohen's d)
- Report exact p-values wherever possible alongside the summary statistics and 95% confidence intervals. These should be reported for all key questions and not only when the p-value is less than 0.05.

Please outline where this information can be found within the submission (e.g., sections or figure legends), or explain why this information doesn’t apply to your submission:

Statistical analysis methods / statistical tests used: Bootstrap measures to assess mean differences are described in the Methods under the subheading, “Bootstrap statistical testing**”**. The statistical test to determine whether the distribution of unit d’ values were shifted from zero (Fig 8a-b) is described in Results (a Wilcoxon sign rank test).

Raw data: our dataset is large and never < 10.

Exact values of N: Values of N (number of sessions or units) are reported throughout the paper, as specified in the text and figure legends.

Definitions of center: means are reported throughout the paper, as specified in the text and figure legends.

Methods of multiple test correction: p-value criterion are clearly described.

Dispersion measures: Computation of confidence intervals are described in the Methods under the subheading, “The single-exposure visual memory task**”**

Exact p-values are reported.

(For large datasets, or papers with a very large number of statistical tests, you may upload a single table file with tests, Ns, etc., with reference to sections in the manuscript.)

**Group allocation**

- Indicate how samples were allocated into experimental groups (in the case of clinical studies, please specify allocation to treatment method); if randomization was used, please also state if restricted randomization was applied
- Indicate if masking was used during group allocation, data collection and/or data analysis

Please outline where this information can be found within the submission (e.g., sections or figure legends), or explain why this information doesn’t apply to your submission:

Our study was not organized into experimental groups. Our study is a comparison of the sensitivities of behavioral performance and neural signals to forgetting (with time). Experimental subjects were two monkeys.

**Additional data files (“source data”)**

- We encourage you to upload relevant additional data files, such as numerical data that are represented as a graph in a figure, or as a summary table
- Where provided, these should be in the most useful format, and they can be uploaded as “Source data” files linked to a main figure or table
- Include model definition files including the full list of parameters used
- Include code used for data analysis (e.g., R, MatLab)
- Avoid stating that data files are “available upon request”

Please indicate the figures or tables for which source data files have been provided:

n/a
